# Axonal activity *in vivo*: technical considerations and implications for the exploration of neural circuits in freely moving animals

**DOI:** 10.3389/fnins.2015.00153

**Published:** 2015-05-06

**Authors:** Jeremy M. Barry

**Affiliations:** ^1^Department of Neurology, Geisel School of Medicine at DartmouthLebanon, NH, USA; ^2^Epilepsy, Development and Cognition Group at UVM, Department of Neurological Sciences, University of VermontBurlington, VT, USA

**Keywords:** axonal activity, *in vivo* electrophysiology, hippocampus, neural conduction, circuits and systems

## Abstract

While extracellular somatic action potentials from freely moving rats have been well characterized, axonal activity has not. We have recently reported extracellular tetrode recordings of short duration waveforms (SDWs) with an average peak-trough duration less than 172 μs. These waveforms have significantly shorter duration than somatic action potentials and tend to be triphasic. The present review discusses further data that suggests SDWs are representative of axonal activity, how this characterization allows for more accurate classification of somatic activity and could serve as a means of exploring signal integration in neural circuits. The review also discusses how axons may function as more than neural cables and the implications this may have for axonal information processing. While the technical challenges necessary for the exploration of axonal processes in functional neural circuits during behavior are impressive, preliminary evidence suggests that the *in vivo* study of axons is attainable. The resulting theoretical implications for systems level function make refinement of this approach a necessary goal toward developing a more complete understanding of the processes underlying learning, memory and attention as well as the pathological states underlying mental illness and epilepsy.

## Introduction

The *in vivo* firing properties of extracellularly recorded hippocampal pyramidal cells and interneurons have been well characterized (Ranck, 1973; Fox and Ranck, 1975, 1981; Henze et al., 2000). Reliable identification of these cell types was essential for the experiments confirming their role in the underpinnings of spatial cognition as well as attention (Kentros et al., 2004; Muzzio et al., 2009; Fenton et al., 2010) and in the generation of network oscillations (Kamondi et al., 1998; Penttonen et al., 1998; Buzsaki, 2002; Colgin and Moser, 2010). Normal neural function and therefore properly ordered cognition, requires the faithful propagation of somatic action potentials between neurons and their downstream targets along axons (Swadlow et al., 1980b; Womelsdorf and Fries, 2007; Kleen et al., 2010; Singer, 2011; Zalesky et al., 2011).

Great strides have been made in the diffusion weighted imaging of axonal processes (Basser and Pierpaoli, 1996), the visualization of axonal projections and anatomical connectivity (Chung et al., 2013), as well as *in vitro* studies examining the details of signal propagation in invertebrate (Bucher and Goaillard, 2011; Ballo et al., 2012) and mammalian axons (Kole et al., 2007; Shu et al., 2007; Yang et al., 2013; Xia et al., 2014). These *in vitro* studies have shown that axons are more than merely reliable conduits for ordered signal propagation and may be directly involved in complex information processing (Debanne, 2004; Bucher and Goaillard, 2011; Bakkum et al., 2013), contribute to high frequency network oscillations (Traub et al., 2003a,b; Gloveli et al., 2005; Dugladze et al., 2012) and possess intrinsic breaking mechanisms that can potentially halt seizure propagation (Meeks et al., 2005). However, the *in vivo* recording of axonal activity in freely moving animals has received little attention since early microelectrode recordings were developed (Amassian et al., 1961; Cooper et al., 1969).

While axonal activity has commonly been deemed inaccessible by standard *in vivo* recording probes, recent data provides a range of waveform properties that allow for more accurate classification of putative axonal activity in freely moving animals (Robbins et al., 2013). The present review describes the parameters of *in vivo* extracellular recordings of short duration waveform (SDW) activity as well as further technical justification for why these waveforms are representative of the propagation of action potentials along axons. The review also discusses how the definition of SDWs and their secondary waveform properties serve to better clarify the boundaries between different classes of extracellularly recorded unit types, particularly putative axons and interneurons. The ability to correctly identify and reliably record axonal activity provides a means of interrogating neural circuits and understanding signal integration between disparate brain regions. Examples of this are discussed with regard to the brain's “space circuit” as well as thalamocortical projections in the generation of specific oscillatory rhythms in cortex. In addition, axonal processes are reviewed that modify activity in neural circuits and may ultimately suggest a complex role for axons in information processing through influencing signal propagation and local field oscillations. Finally, several future directions are suggested regarding possible experiments and the technical challenges that must be overcome in order to bridge the gap between *in vitro* and *in vivo* recording methodologies. Furthering this approach will allow for the more systematic interrogation of axonal action potentials toward larger questions of the role of axons in cognitive and pathological processes in freely moving animals.

## Are short duration spikes representative of axonal activity?

Robbins et al. (2013) demonstrated that it is possible to monitor axonal activity in white and gray matter and to simultaneously record ensembles of cells and axons using conventional tetrodes in awake, freely moving rats. Robbins et al. also proposed that SDWs represent the propagation of action potentials along axons and described three common electrophysiological features of SDWs: The primary feature, as indicated by the name given to these units, is that they are significantly brief. SDWs have a mean peak-trough duration of 172 μs. A secondary feature exhibited by SDWs is that they tend to be triphasic (a brief hyperpolarization phase, a longer-duration depolarization phase, and another brief hyperploarization phase). Both of these features have been associated with extracellular recordings of action potentials in axons *in vitro* (Raastad and Shepherd, 2003; Kole et al., 2007; Bakkum et al., 2013). Recording from cultured neurons on an electrode microarray, Bakkum et al. (2013) noted that the waveforms of propagating action potentials at different sites along the same axon were largely triphasic, with a notable positive first phase, or biphasic. The authors speculate that this subtle difference in waveform shape could be due to differences in morphology and the variation of ion channels at each recording site.

With regard to spike duration, Kole et al. (2007) found a significant and progressive decrease in the duration of action potentials from the soma through the extent of the axon in layer 5 pyramidal cells. The half-width of the action potential decreased as the patch recording sites moved from the soma (503 ± 7.4 μs), to the most distal region of the axon initial segment (290 ± 18.8 μs). Finally, intracellular patch recordings from the cut end of distal axons, or axon blebs (Hu and Shu, 2012), up to 720 micrometers from the axon hillock had the shortest half-width (266 ± 8.5 μs). The first order derivative of intracellular axon bleb values match the duration of extracellularly recorded SDWs shown in Robbins et al further suggest that SDWs are not somatic action potentials and are likely representative of action potential propagation along axons. With regard to the mechanism by which somatic action potential duration is compressed during signal transmission from the soma, Kole et al. (2007) propose that Kv1 channels strategically positioned in the axon initial segment decrease the duration of the axonal action potential waveform and allow for the integration of slow subthreshold signals. In this manner the Kv1 channels are able to control the presynaptic action potential waveform and synaptic coupling in local circuits.

**Figure 1:**
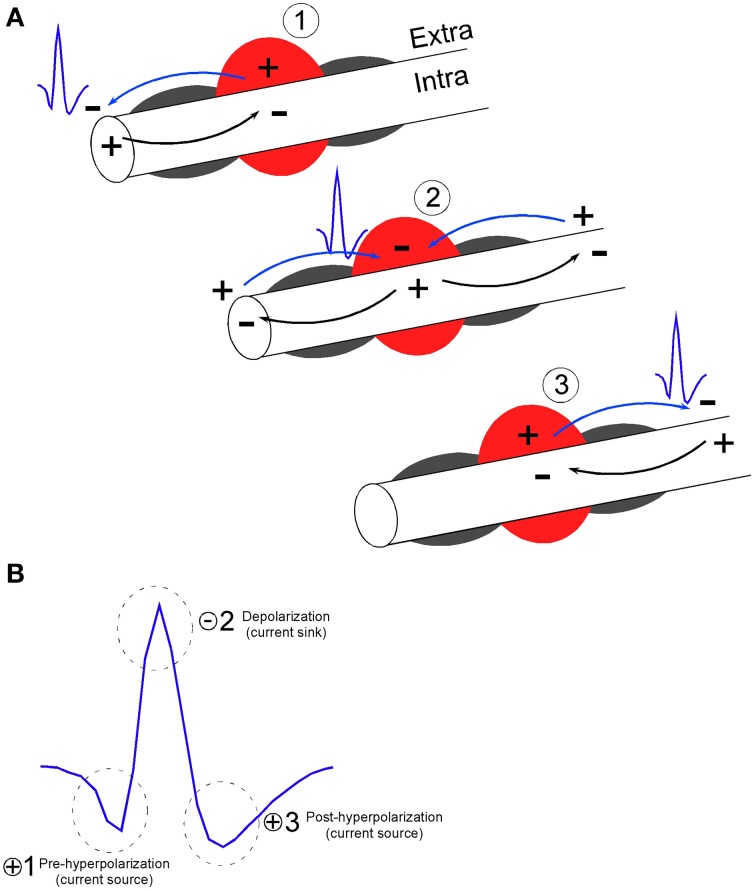
**Model of extracellular recording of action potential propagation along axons (Johnston and Wu, 1995) resulting in the triphasic shape of short duration waveforms. (A)** At the point of action potential initiation on the left, local current will flow into the axonal membrane (Intra) and provide a current sink, while the point where the current exits the axonal membrane (Extra) provides a current source. If recordings are made near myelinated axons, current flow is likely generated at the nodes between myelinated segments (gray semi-circles surround the axon). Relative to a distant ground or reference electrode, the current sink generates the negative voltage potential while the source generates the positive voltage potential. As the propagating action potential passes the region of stationary electrode, (denoted by the red circle) the location of sinks and sources will change with time and alternate between a source (1), a sink (2) and a source (3). **(B)** The electrode will therefore record a triphasic waveform composed of a positive voltage potential indicating membrane hyperpolarization (1), a negative potential indicating membrane depolarization (2), and a positive voltage potential indicating membrane hyperpolarization (3). In reference to the depolarization phase (2), phase 1 and 3 are referred to as the pre and post hyperpolarization phase, respectively. The waveform therefore represents the movement of current flow in and out of the axonal membrane during the duration of the action potential. Example waveform has peak-valley duration of 0.150 ms and signal to noise ratio of 3:1.

The Robbins et al. description of axonal activity also matches that of classic fiber tract recordings using 3 μm diameter tungsten wires set in micropipettes. With regard to recordings of brief units, Mountcastle preferred to refer to putative cortical stellate neuron waveforms as “thin spikes” due to the uncertainty that they may have been thalamocortical fibers (Mountcastle et al., 1969). Both Amassian et al. (1961) and Cooper et al. (1969) reported recordings of brief, triphasic action potentials approximately 130 μs in duration in a variety of species (cat, squirrel, monkey) and recording locations (optic tract, geniculostriate fibers in the visual cortex, pons and medulla, and also the cuneate nucleus). The triphasic waveform shape of putative axonal recording is common to these descriptions of axonal recordings as well as descriptions of SDWs in white matter tracts in Robbins et al. As described in Figure 1, the triphasic waveform shape arises when a propagating action potential is elicited near the recording electrode. At this point of initiation, local current will flow into the axon and provide a current sink, while the point where the current exits provides a current source. Relative to a distant ground or reference electrode, the sink generates the negative potential while the source generates the positive potential. As the propagating action potential passes the stationary electrode, the location of sinks and sources will change with time and alternate between a source, a sink and return to a source. The electrode will therefore record a triphasic waveform composed of a positive potential, a negative potential and a final positive potential representing the movement of current flow in and out of the axon during the duration of the action potential (Johnston and Wu, 1995).

KEY CONCEPT 1 Electrical activity recorded from putative axons differs from somatic activity by its waveform shape that is characteristically brief and tends to be triphasic. Short duration waveforms (SDWs) may represent the propagation of action potentials from a somatic source.

It is still unknown as to how the presence or absence of myelination along axons at the point of contact with the electrode might affect waveform shape (Robbins et al., 2013). This is a relevant consideration as mammalian white matter tracts such as the corpus callosum typically have a mix of both myelinated and unmyelinated axons (Waxman and Swadlow, 1976; Sturrock, 1980; Swadlow et al., 1980a) and neocortical pyramidal cells tend to have myelinated axons that are interspersed with long unmyelinated tracks (Shu et al., 2007; Tomassy et al., 2014). Myelin has a high resistance and the total charge movement involved in the propagation of an action potential in a myelinated fiber should be less than in a non-myelinated fiber as the extracellular current density is greater close to the nodes of a myelinated fiber. For this reason, one may assume that if extracellular recordings are performed along myelinated axon tracts, the signal will primarily represent extracellular ion flow near or between nodal sites (see Figure 1). Future studies involving recordings of axonal activity *in vivo* may address the effects of myelin on extracellular waveform properties through the pharmalogical separation of myelinated and unmyelinated axons. For example, *in vitro* studies of axons in the rat corpus callosum have suggested the dissociation of myelinated and unmyelinated components of compound action potentials through the local application of the potassium channel blocker 4-amino-pyridine (4-AP) (Swanson et al., 1998). Central unmyelinated axons have 4-AP sensitive potassium channels along their extent while myelinated axons only have 4-AP sensitive potassium channels at the nodes of Ranvier (Preston et al., 1983). Therefore, 4-AP had no effect on the myelinated axon component of the compound action potential while it significantly increased the duration of the unmyelinated component (Swanson et al., 1998).

In agreement with descriptions of SDWs in Robbins et al. early reports of axonal action potentials also reported that the amplitude and shape of axonal recordings were stable for at least for up to 24 h (Amassian et al., 1961). While the largest amplitude axonal spikes reported by Cooper et al. (1969) were ~150 μV, Robbins et al. reported recordings of several axons that were significantly larger in amplitude, particularly in the alveus. One can assume that the amplitude of axonal action potentials will increase when the recording electrode is closer to the axonal membrane. However, whether or not the presence of myelin places a ceiling effect on the amplitude of extracellularly recorded axonal action potentials remains to be seen.

The third defining feature, in the case of tetrode recordings, is that the activity of SDWs generally appears to be restricted to a single wire and is therefore suggestive of a much smaller source area as compared to activity recorded in the area of the soma. However, this feature may be region dependent. It has been reported that in the ventrobasal thalamus, within a reasonably small volume of neuropil (<20 μm), close to 100 terminals of the same axon branch emanating from the thalamic reticular nucleus can be present. Since the terminal clusters fire together close in time, it is predicted to generate a large enough extracellular field response that can be recorded along several contact points of the same silicon recording probe (Bartho et al., 2014). The larger implication of this study, as well as Robbins et al. (2013), is that existing protocols for the extracellular recording of somatic action potentials in freely moving animals (Chang et al., 2013) appear to work just as well for the extracellular recording of axonal action potentials.

**Figure 2:**
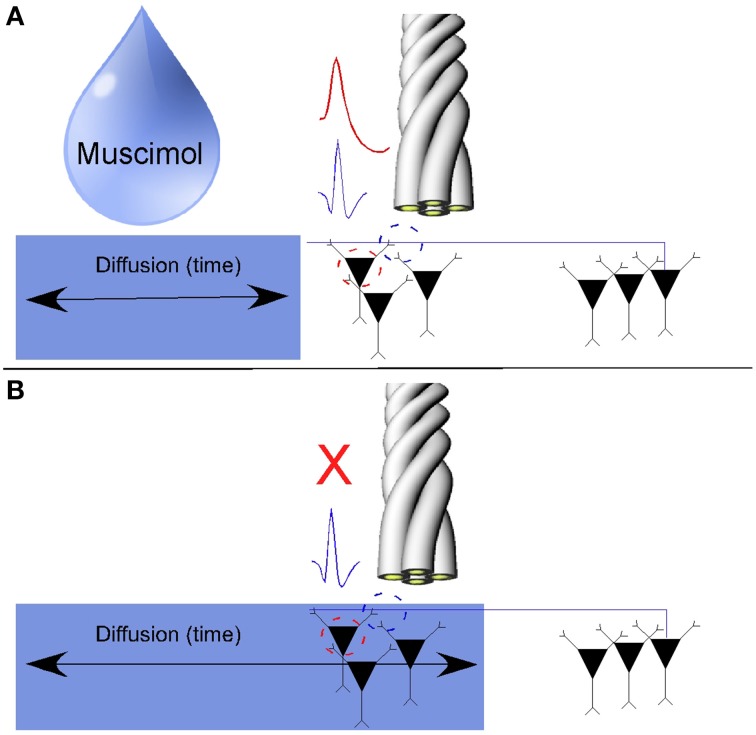
**Pharmacological separation of axonal activity from somatic activity using the GABAa agonist Muscimol. (A)** Somatic action potentials and axonal activity were simultaneously on the same tetrode. **(B)** Somatic action potentials were inactivated over time as muscimol diffused through the hippocampus. As axons tend to have a sparse density of GABAa receptors, axonal activity persisted while simultaneously recorded cells on the same tetrode were inactivated (Adapted with permission from Ramón y Cajal, 1953).

Robbins et al. (2013) also showed that, in hippocampal gray matter recordings, a local injection of Muscimol near the recording tetrodes inactivates somatic action potentials while axonal action potentials persist, at least in the first couple of minutes after somatic inactivation. While the role of GABA_A_ receptors along axons continues to be studied, several reports suggest that the activation of GABA receptors along the extent of the axon proper is not sufficient to arrest the orthodromic propagation of axonal action potentials (Dugladze et al., 2012; Xia et al., 2014). A schematic of the muscimol injection experiment is shown in Figure 2.

**Figure 3:**
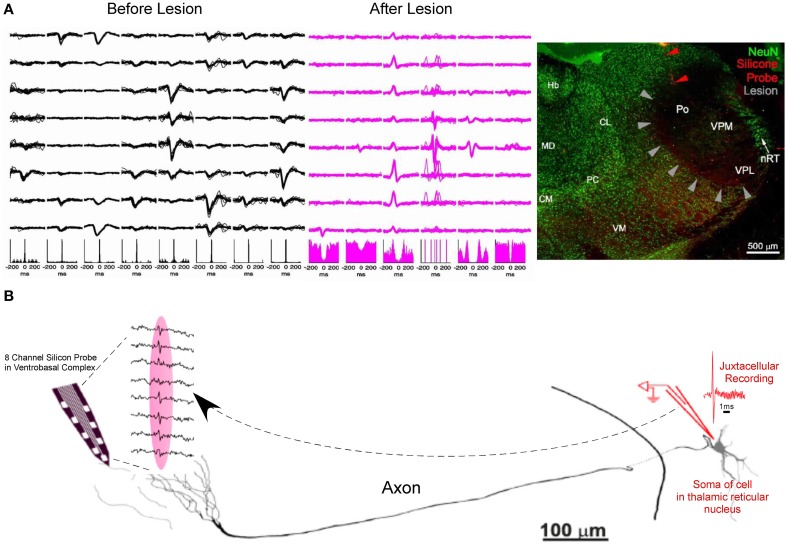
**Further evidence of short duration spikes as originating from the propagation of action potentials along axons. (A)** Spike waveforms of 8 different units along the 8 recording sites of a silicon probe (Left). Kainic acid is focally injected into the ventrobasal thalamus, in the vicinity of the recording probe. As kainic acid selectively lesions the soma and leaves axonal projections intact, somatic actional potentials are absent from the recording, leaving only axonal activity in the same recording space (middle) and absence of spindle modulation in the corresponding autocorellograms. NeuN staining exhibits lesioning of thalamocortical cells in the ventrobasal thalamus (gray arrowheads) in the vicinity of the recording site (Red arrowheads) (Right). **(B)** Camera lucida drawing of a juxtacellularly recorded and labeled reticular neuron (spike shown in red) with simultaneous extracellular recording of its axonal arbor in the ventrobasal thalamus (traces from 8 recording sites are shown between dashed lines). Juxtacellularly recorded action potentials from the soma were time-locked with probe recordings from the axons with a conduction delay of less than 0.5 ms (Adapted with permission from Bartho et al., 2014).

An experiment with a similar logic aimed toward separating somatic and axonal action potentials was recently shown by Bartho et al. (2014). They simultaneously recorded in the ventrobasal thalamus with silicon shanks while using iontophoresis to deliver the axon-sparing neurotoxin kainic acid into the ventrobasal thalamus, thereby lesioning local soma while preserving axons. Four hours after the delivery of kainic acid, previously recorded somatic action potentials were gone. In the absence of somatic activity, only axonal activity remained which was no longer locally modulated by spindles (waxing and waning field potentials at 7–14 Hz). The absence of cells in the vicinity of the recording probe was then confirmed by NeuN staining (Figure 3A). To further the case that the axonal activity in the ventrobasal thalamus were projections from the reticular nucleus, the authors again recorded in the ventrobasal using silicon probes while simultaneously performing juxtacellular recording and labeling in the reticular region. The somatic action potentials of the juxtacellularly recorded reticular neuron were time locked (<0.5 ms) to the extracellularly recorded axonal activity in the ventrobasal thalamus. Morphological reconstruction of the juxtacellularly recorded cell confirmed its identity as a reticular neuron with axonal projections close to the vicinity of the track occupied by the recording probe (Figure 3B).

KEY CONCEPT 2 The ability to record axons using tetrodes and silicon probes has been suggested through the use of: (1) Exploiting pharmacodynamic differences between putative axons and soma. (2) The use of excitotoxic drugs that selectively lesion the soma while leaving axonal projections intact. (3) Simultaneous juxtacellular recording of the somatic source with extracellular recordings of a distal axonal arbor.

## Classification of interneuron vs. axon

As previously mentioned, the electrophysiological parameters of hippocampal pyramidal cells and interneurons have been well characterized (Ranck, 1973; Fox and Ranck, 1975, 1981; Henze et al., 2000) in terms of their waveform and firing properties, where interneurons tend to have a SDW than pyramidal cells. The main reason for this difference in spike duration between these different cell types encompasses both morphology and the density of ion channels that determine ion conductance (Lai and Jan, 2006). The expression of potassium channels has been found to play a critical role in determining spike duration. In particular, the unique properties of Kv3.1 and Kv3.2 channels enable sustained high frequency firing in fast-spiking interneurons by minimizing the duration of the hyperpolarization period and thereby facilitating the recovery of Na channel inactivation (Erisir et al., 1999).

These properties of neuronal cell types are part of the essential tool kit for neuroscientists who perform *in vivo* electrophysiology as without visual access to the tissue at the tip of the electrode or silicon probes, there is a need to infer the identity of each cell type. As mentioned in Robbins et al. (2013), there is a danger in relying on individual waveform features such as half-width or spike duration. While the average interneuron peak-trough duration was significantly different from axonal activity, the interneurons can overlap with the upper range of axons as the peak trough-duration of some of the interneurons in the Robbins et al. data set were approximately 200 μs. Bartho et al. (2004) also reported putative interneurons that fell within the range of axons.

While the distinction between axon and interneuron peak-trough duration may be sufficiently problematic, there is data that indicates variability of spike duration in pyramidal tract neurons (PTNs) in primate motor cortex. Pyramidal neurons with large soma tend to have larger axons with faster conduction velocity that ultimately leads to shorter spike durations that range from 0.15 to 0.45 ms (Vigneswaran et al., 2011). While this study is important as it shows how underlying physiology can create variability in waveform features within macaque cortex, it also points out how these duration values overlap with those reported for putative inhibitory interneurons as well as putative axons recorded from rodents (Fox and Ranck, 1981; Henze et al., 2000; Robbins et al., 2013). Ultimately, the most important lesson from Vigneswaran et al. is that spike duration alone is not the best indicator of cell type. If possible, firing properties or secondary waveform characteristics should also be used to indicate whether the isolated unit is an excitatory neuron, inhibitory neuron, or putative axon.

**Figure 4:**
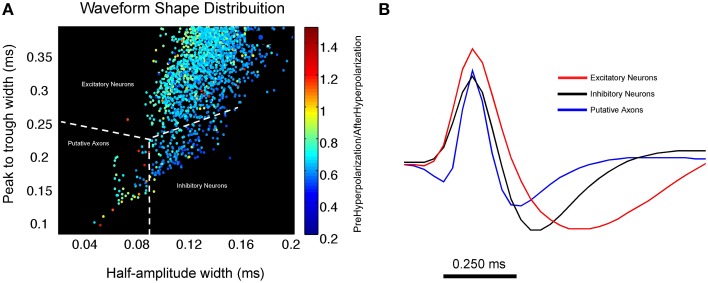
**Using waveform shape distributions to cluster excitatory, inhibitory and putative axonal activity in the medial prefrontal cortex. (A)** Scatterplot of waveform distribution that plots peak-trough width against half amplitude width. Each data point represents an individual cell waveform that is color coded to reflect its pre-hyperpolarization and post-hyperpolarization ratio. The use of secondary waveform features in conjunction with spike duration allows for the reliable separation of 3 distinct clusters of excitatory and inhibitory neurons as well as putative axons. Inhibitory neurons tend to exhibit a larger half amplitude width as well as a lower pre-hyperpolarization/post-hyperpolarization ratio (shown in blue) when compared to putative axons. **(B)** Average waveform for all units in each cluster; excitatory neurons are shown in red, inhibitory neurons are in black, putative axonal activity is shown in blue. Average waveform of putative axons strongly resembles those described in Robbins et al. (2013) (Figure compliments of Dr. Nathan Insel; Dataset from Insel and Barnes, 2014).

One example of a recent advance toward disambiguating excitatory and inhibitory neurons, as well as putative axons, involved not only taking spike duration into account but to also utilize comparisons of hyperpolarization periods before and after the depolarization period. In an effort to decode circuit operations in the medial prefrontal cortex during behavior, Insel and Barnes (2014) employed this method to separate excitatory neurons, inhibitory interneurons and putative axonal activity (Figure 4). By plotting the peak-trough width against the half-amplitude width, and further color-coding each data point by the ratio of its pre and post-hyperpolarization, they were able to more accurately cluster projection neurons, inhibitory neurons and putative axonal fibers of passage. This process allowed for the more accurate characterization of how excitatory neurons provide information about behavioral context as well as reward sites while inhibitory neurons are most associated with movement and sensory stimulation. This relationship was also found to exist between adjacent neurons with reciprocal inhibitory-excitatory connections (Insel and Barnes, 2014). The putative axons described by Insel and Barnes were found to have waveforms similar in both duration as well as waveform shape to those described by Robbins et al. (2013) and serve as a strong example of how secondary waveform features in conjunction with spike duration improve upon boundaries used to classify extracellularly recorded units and allow for the reliable separation of putative axons and interneurons. Omitting putative axons in this case was desirable as it allowed for a more conservative analysis between inhibitory and excitatory neuron classes in the prefrontal cortex.

## Understanding signal integration through axonal activity

**Figure 5:**
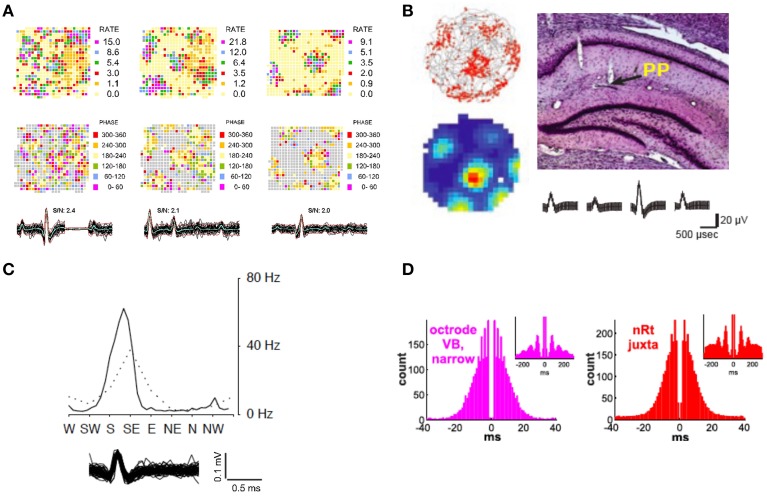
**Exploring neural circuits through the *in vivo* extracellular recording of axonal activity. (A)** Firing rate maps of 3 axons recorded in the alveus layer of the hippocampus display grid firing patterns (Top Row). Each axon appears to be organized by local theta oscillations (Middle Row) as spike activity advances from late to early phases of the local theta cycle as the animal passes through each grid vertex. The overlay of 60 spikes as well as the average waveform for each axon is displayed for each wire (Bottom Row) (Adapted from Robbins et al., 2013). **(B)** Regular grid patterns are found from a putative axon in the termination area of the hippocampal peforant path (Top Right). Trajectories (in gray) with spike locations (red dots), as well as color-coded rate maps (red is maximum rate, blue is minimum, i.e., 0 Hz) (Top and Bottom Left respectively. Average waveform for putative axon (Bottom Right) (Adapted with permission from Leutgeb et al., 2007). **(C)** Head direction tuning exhibited by a putative axon in the hippocampus. The peak axonal activity occurs at apklpproximately 60 Hz when the animal's head is faced between S/SE (Adapted with permission from Leutgeb et al., 2000). **(D)** Autocorrelograms of an axon recorded in the ventrobasal thalamus using a silicon probe and a juxtacellularly recorded and labeled reticular neuron. The plots exhibit the longer bursts and spindle modulation (see insets) of reticular neurons that distinguish them from thalamocortical neurons in the ventrobasal thalamus (Adapted with permission from Bartho et al., 2014).

Robbins et al. (2013) also reported the capture of several axons in the alveus layer of hippocampus, a border structure composed of axons, whose activity displayed a distinctive pattern resembling repeating equilateral triangles (Figure 5A) typically associated with grid cells (Hafting et al., 2005; Boccara et al., 2010). Some alvear axons carry afferent input from more distant brain regions such as the entorhinal cortex (Deller et al., 1996; Brun et al., 2008). The axonal activity reported by Robbins et al. could originate in the entorhinal cortex, the presubiculum, or parasubiculum (Hafting et al., 2005; Boccara et al., 2010). Grid cells from layer II of the medial entorhinal cortex show phase precession (O'Keefe and Recce, 1993; Huxter et al., 2003) in that their spike activity advances from late to early phases of the theta cycle as the animal passes through a grid vertex (Hafting et al., 2008). A similar organization of axonal grids by local theta oscillations was also found to be present. Averaged across the entire 16 min session, the spiking of the SDWs while the rat is in the central part of each field tends to occur between 180°and 240° of the theta cycle while firing at the periphery of each field tends to be greater or less than that range in the theta cycle. Similarly, grid firing has also been reported in the axon terminals of both the medial and lateral aspects of the perforant path (Figure 5B), white matter projections that serve as the principal input source from the medial entorhinal cortex to the hippocampus (Leutgeb et al., 2007). The waveforms of these units also meet the criteria for axons described in Robbins et al. (2013) as they have similar spike durations and have the same triphasic waveform.

In another case of the convergence of spatial information in the hippocampus, the firing of putative axonal activity in hippocampus has been shown to possess head direction properties (Leutgeb et al., 2000), or selective tuning discharge when the animal's head is pointing in a specific direction. Head direction signals are found in the anterior dorsal thalamic nucleus (Taube, 1995), lateral mammillary nucleus (Wiener, 1993), lateral doral thalamic nuclei (Mizumori and Williams, 1993), the striatum (Wiener, 1993) as well as the retrosplenial cortex (Cho and Sharp, 2001) and medial enthorinal cortex (Chung et al., 2013). However, of these structures, only the medial entorhinal cortex has direct monosynaptic input into the hippocampus (Taube, 2007). Histology from Leutgeb et al. (2000) indicates that several of these head direction units were recorded near white matter tracts in the region of the alveus as well as in the end terminals of the perforant path (Figure 5C). Coupled with the brief, triphasic waveform of many of these units, one may assume that these were fibers of projection from head direction cells from the medial entorhinal cortex.

As both Leutgeb et al. (2000, 2007) suggest, the combination of both head direction and grid signals in the hippocampus would allow for strong synaptic interactions which could integrate these spatial signals in the generation of the hippocampal representation of space. That it is now apparent that recordings of axonal activity in freely moving animals are now accessible, it may lead to new thinking regarding the integration of multiple streams of converging spatial information in the generation and alignment of hippocampal place fields and ultimately the cognitive map (O'Keefe and Nadel, 1978). Strengthening this argument, a viral vector (recombinant adeno-associated virus) has been developed that induces expression of light-sensitive transgenes into local hippocampal neurons as well as neurons in the medial entorhinal cortex through retrograde transport along their hippocampal projecting axons (Zhang et al., 2014). This technique has allowed for studies of functional connectivity of the entorhinal-hippocampal “space circuit” and the interplay between place cells in the hippocampus and the different functional cell types in the medial entorhinal cortex (grid cells, head direction cells, conjunctive cells, and border cells) (Witter and Moser, 2006). This approach, combined with the simultaneous recording of axonal activity in the hippocampus and the origins of these axons in the medial entorhinal cortex may also shed new light on the phenomenon of phase precession (O'Keefe and Recce, 1993). It remains to be seen if action potentials from the entorhinal cortex arrive at their end terminals in the hippocampus, such as in the stratum lacunosum-moleculare at the same phase of theta as their somatic source and if phase therefore plays a role in their synaptic integration with hippocampal target neurons. It is also unknown whether there is a gating mechanism at the synaptic contacts between the terminals of entorhinal inputs and the dendrites of hippocampal pyramidal cells that controls which type of spatial information, for example from grid or boundary cells, is selectively projected to the hippocampus depending on spatial demands.

A final example of the convergence of 2 different streams of information, between local cells and their contact with local axons can be seen in Bartho et al. (2014). Their anatomical and physiological data clearly showed that simultaneous measures of somatic action potentials from thalamocortical cells and the axonal activity stemming from cells in the reticular nucleus, from the same recording probes, are measures of reciprocally coupled excitatory ventrobasal and inhibitory reticular cell populations. Further evidence of this can be seen in Figure 5D, where the firing properties of axonal activity in the ventrobasal thalamus strongly resembles the firing properties of juxtacellularly recorded reticular cells. In contrast to the ventrobasal thalamocortical cells, these have much longer bursts and more spindle modulation. The assertion that “narrow” spikes in the ventrobasal were axonal projections from the reticular nucleus allowed for the quantification of cycle-by-cycle alterations in their firing in relation to the “wide” spikes of local thalamocortical cells *in vivo*. Ultimately, Bartho et al. showed that spindles with different lengths were coupled with distinct reticular activity, and reticular firing dropped sharply before the end of all spindles. While initial reticular and thalamocortical activity was correlated with spindle length, the reticular correlation was found to be stronger. This gives us important insights into the mechanisms controlling the duration and termination of an oscillatory event that is critical to stage 2 sleep as well as normal brain function.

KEY CONCEPT 3 Spikes that propagate along fibers of passage represent the movement of information between distal brain regions and serve as an illustration of neural circuits underlying network processes in the brain.

## Beyond somatocentrism: are axons more than just cables?

Harkening back to the beginning of modern neuroscience, Cajal first defined the axon as a long process devoted to the faithful transmission of neuronal information from the soma to the nerve terminal (Ramón y Cajal, 1953). While there is a great deal of data to suggest that axons do act as cables, as implied in the previous section, they may be capable of much more. A growing body of literature suggests that axons possess functional and computational abilities beyond signal propagation that may attribute them with a significant role in information processing. The suggested mechanisms underlying these abilities are decreased conduction or reflection at branch points, axonal geometry, and spike timing control by voltage gated ion channels (Debanne, 2004). Most of the relevant notions regarding the computational abilities of axons come from computer models (Segev and Schneidman, 1999) or data from invertebrate neurons (Ballo and Bucher, 2009; Ballo et al., 2010, 2012; Bucher and Goaillard, 2011). As most of our understanding of propagation in mammalian axons comes from *in vitro* preps, very little is known about axonal activity in functioning systems.

Perhaps the most robust mechanism that has been proposed as a mechanism of putative information processing in the axon is that of propagation fidelity between the soma and the distal axon (Meeks et al., 2005; Bucher and Goaillard, 2011). In a slice preparation, using whole cell recordings from CA3 pyramidal cells and extracellular axons up to 0.8 mm from the soma, Meeks et al. (2005) showed that axonal action potentials were more resistant to amplitude reduction than their corresponding somatic spikes. They found that increasing extracellular potassium caused relatively small decreases in the amplitude of axonal spikes. However, if the same conditions were repeated during prolonged epileptiform spiking in the soma, the amplitude of axonal spikes was found to decrease significantly. While the obvious importance of this result is that it suggests that axons have an endogenous brake on the propagation of seizures, it also shows that potassium currents react to recent sustained activity in a manner that leads to changes in axonal membrane potential that act as a check to signal propagation. While results like Meeks et al. (2005) suggest that axons may act in a manner similar to high pass filters, there are also important technical considerations in terms of intracellular and extracellular recording. In finding results contrary to those of Meeks et al. (2005), Shu et al. (2007) conducted extracellularly and whole-cell recordings of axonal action potentials. They found that action potentials recorded extracellularly could suffer from a significant signal to noise problem. During epileptiform bursts axonal spikes may become smaller than background noise levels in extracellular recordings leading to many of these action potentials being undetected. However, propagation failures have also been reported in the axons of rodent Purkinje cells *in vitro* where the soma was recorded in whole-cell configuration and axonal activity was recorded in cell-attached mode up to 0.8 mm from the soma (Monsivais et al., 2005). While action potential propagation was reliable at spike frequencies below 200 Hz, propagation failures were observed above 250 Hz. Moreover, complex bursts from Purkinje cells were not found to reliably propagate along the axon. Typically, only the first and last spike of a complex burst were found to propagate reliably. This complex spike failure rate was found to be strongly influenced by membrane potential and tended to occur when the cell body was depolarized (Monsivais et al., 2005).

Considering that spike propagation in most cases involves interactions between various ionic conductances exhibiting gating properties with several different time constants, which are also affected by summating pump currents or intracellular calcium dynamics, it is probable that most axons will also exhibit a sort of short-term hysteresis with regard to conduction velocity (Bucher and Goaillard, 2011). These sorts of signal alterations during propagation will undoubtedly affect the precise temporal structure of spikes at their ultimate targets. Ultimately, low temporal fidelity between soma and target may be good enough for information processing in terms of rate coding. In fact, this sort of “lossy” propagation with respect to complex rates of signaling may be an important feature of the encoding process.

Apart from the effects of recent activity on the temporal fidelity of action potential propagation from proximal to distal sites along the axon, there may also be a strong influence of neuromodulators on the waveform shape of propagating axonal action potentials (Yang et al., 2013). Through simultaneous whole-cell recording at the soma and axon blebs in layer 5 pyramidal neurons in rat prefrontal cortex, Yang et al. have shown that the spike duration of axonal action potentials increases significantly following exposure to D1 receptor dopamine agonists. The study also found the effects of dopamine agonists on axonal action potential waveforms in axons that were disconnected from the soma, suggesting that there are dopamine receptors on the axon trunk that could have modulatory effects on axonal potassium channels. The broadening of axonal action potential waveforms may enhance synaptic transmission and provide a potential mechanism for presynaptic membrane potential-dependent communication between neurons (Yang et al., 2013). Ultimately, this mechanism may fine tune the neuronal and circuit activity in the prefrontal cortex and imply a further role for dopamine in both neural coding schemes and attentional processes (Nieoullon, 2002).

It remains to be seen whether or not dopaminergic neuromodulation of fibers of passage may underlie attention-related rate firing phenomena found in hippocampal pyramidal neurons. One such example of this is overdispersion, or the inherent variability of place cell firing in rats (Fenton et al., 2010). Without behavioral demand, place cells show significantly more variability in their firing than during a more attentive state in which animals are asked to perform spatial tasks. Another example may be the characteristic firing field instability typical of inattentive mice (Kentros et al., 2004). Kentros et al. showed that mice that performed a complex spatial operant task tended to have increased firing field stability between extended recording sessions then those that did not have to perform the task. Moreover, firing fields were also found to be more stable following systemic injections of dopaminergic agonists, furthering the link between dopamine in relation to attention and its broad effects on network states.

Finally, the morphological properties of axons imbue them with a prodigious influence over network properties in the hippocampus (Gloveli et al., 2005; Dugladze et al., 2012). For example, interneurons projecting from the oriens layer to lacunosum moleculare (O-LM cells) exhibit a 2-fold larger axonal spread in the longitudinal plane of the hippocampus rather than its transverse plane. This complex axonal arbor may therefore provide much stronger theta frequency patterned output to the distal segments of pyramidal cells and to interneurons in the longitudinal plane. This organizational feature of the hippocampus allows distributed activity between distinct layers to be temporally co-ordinated at theta oscillations (Gloveli et al., 2005). In addition, axonal processes have a strong reaction to gamma oscillations, *in vitro*. Following gamma induction, ectopic action potentials occurred at a high frequency in the distal axons of hippocampal pyramidal cells in layer CA3. Axo-axonic interneurons, that inhibit the axon initial segment of pyramidal cells, play a key role in preventing the antidromic or back propagation of these action potentials into the pyramidal cell's somatodendritic compartments. The interplay between axo-axonic interneurons and the axons of pyramidal cells and the maintenance of inhibition, may play a strong role in the generation of the gamma signal and thereby maintain a functional polarization of pyramidal cells during dynamic network alterations (Dugladze et al., 2012).

During pathological network states, such as during epileptiform activity, all action potentials exhibited an earlier onset in the proximal axon than the soma. The timing of epileptiform spikes was consistent with the generation of ectopic action potentials in axonal compartments approximately 45 μm from the soma (Shu et al., 2007). Importantly, the results of Shu et al. also show that in the initial phase of the paroxysmal depolarization shift (PDS) associated with epileptiform bursts, the number of action potentials generated in the proximal and distal axon was often greater than in the soma. These results are complimentary to Dugladze et al. (2012) in that they imply that action potentials are indeed capable of being generated in the axon. The role played by axo-axonic interneurons in regulating these ectopic action potentials and preventing their penetration of the soma is therefore relevant to studies of epilepsy and the propagation of epileptiform spikes. The role of GABA receptors in the axon in arresting antidromic ectopic action potentials, while allowing orthodromic action potentials, is still not yet well understood. Adding to this complexity, *in vitro* studies using multi electrode arrays along the axon also show that pharmacological blockade of GABA_A_ receptors can also increase the velocity of antidromic action potentials (Bakkum et al., 2013).

KEY CONCEPT 4 Axons are more than cables. Intrinsic axonal properties in combination with local neuromodulators control the level of conduction fidelity between soma and terminal region of the axon and have implications for coding mechanisms in the brain. Axonal processes also allow for the synchronization of disparate brain regions and rhythmicity. These processes are fundamental for normal cognition and understanding mental illness and diseases such as epilepsy.

## Bridging the gap

Convergent evidence from different studies suggest the recording of axonal activity from freely moving animals is not only possible (Robbins et al., 2013; Bartho et al., 2014) but can further our understanding of how the nervous system fosters an array of complex phenomena that underlie cognition. The interrogation of axonal action potentials in freely moving animals allows for the “online” integration of signals between disparate brain regions. For example, exploration of the axonal integration of the “space circuit” in freely moving rats could explain how the hippocampal intersection of different spatial signals from a number of brain structures result in the immergence of a cognitive representation of space (O'Keefe and Nadel, 1978; Witter and Moser, 2006). Moreover, *in vitro* electrophysiological phenomena associated with axonal activity should carry over to further study in freely moving animals. An illustration of this would be the recording of axonal activity in the prefrontal cortex during task performance (see Insel and Barnes, 2014) in order to examine the neuromodulatory effects of dopamine on waveform features such as spike duration (Yang et al., 2013). While technically very challenging, an additional example would be the simultaneous recording of somatic and axonal action potentials in freely moving animals that would test for a possible relationship between propagation fidelity and the variability of pyramidal cell firing rates such as overdispersion in relation to states of attention (Fenton et al., 2010).

Before we can pursue such studies, how do we bridge the gap between the levels of complex functioning networks available in *in vivo* recording in freely moving animals and the level of control and detail available in *in vitro* recordings? One notion is to carry out *in vitro* recording studies using similar materials to those used in *in vivo* experiments such as low impedance micro-wire, as there has been no systematic, in depth study relating morphologically identified axons to recordings from different types of electrodes. Such a study could link the two methodologies and be carried out with existing slice techniques. This would allow for the determination of many different parameters required for the successful isolation of axonal activity such as electrode diameter and impedance levels. With the benefit of visual guidance, the effects of the electrodes point of contact along axonal regions identified as myelinated or unmyelinated might also be determined.

Beyond the use of standard sharp electrodes, laser excitation of voltage sensitive dyes has shown to allow for the *in vitro* measurement of membrane potential transients in submicrometer spatial resolution and sub-millisecond temporal resolution. Data resulting from use of this technique also shows compression of the action potential duration between the soma, axon initial segment and distal axon (Popovic et al., 2012). While this technique cannot be carried out *in vivo*, genetically coded voltage indicators have successfully measured membrane potential in visual cortex of awake mice (Carandini et al., 2015) and hold promise for the visualization of axonal action potentials in the near future.

There is also a need for systematic computational study with regard to extracellular recordings from different varieties of unmyelinated and myelinated axons.

As so little data is available about the biophysical properties of axonal membranes, internal resistivity and channel distribution, carrying out such simulation studies remain a complex undertaking. While already representing a wide range of parameters (Segev and Schneidman, 1999), current models explore the site and threshold of action potential initiation, propagation speed in unmyelinated and myelinated axons, propagation through branch points and other geometrical heterogeneities. They also consider the interaction between axonal morphology and passive and active membrane properties in determining the speed and reliability of action potential propagation (Graham, 2014).

While immensely challenging, overcoming these obstacles will allow for greater understanding of the role axons play in the greater gestalt of coding mechanisms in the brain. By moving beyond somatocentrism in neuroscience we may develop a more complete understanding of network properties underlying learning, memory and attention as well as the pathological states underlying mental illness and epilepsy.

### Conflict of interest statement

The author declares that the research was conducted in the absence of any commercial or financial relationships that could be construed as a potential conflict of interest.
